# Acknowledgment of Invited Editors

**DOI:** 10.1128/mBio.02860-18

**Published:** 2019-02-05

**Authors:** Arturo Casadevall

**Affiliations:** a Bloomberg School of Public Health, Johns Hopkins University, Baltimore, Maryland, USA

## EDITORIAL

On behalf of the editors of *mBio*, I gratefully acknowledge the following individuals, who served as invited editors for the journal in 2018. While not members of the Board of Editors, invited editors serve an important role in the review process. Invited editors are experts in their fields of research who add an additional level of quality to the review process. An editor may assign a paper to an invited editor when he/she would like to have an additional expert opinion of the reviews or when the subject area falls outside the editor’s primary area of expertise.

The time and effort of the following experts in handling articles have been essential to ensuring the high quality of our publications, and their help is greatly appreciated.

John M. Archibald
Martin Fabian Bachmann
Fabio Bagnoli
Vanessa Bailey
Fernando Baquero
Sonia L. Bardy
Frederic J. Barras
Thomas Bernhardt
Elisabeth M. Bik
Steven R. Blanke
Jesse D. Bloom
Daniel R. Bond
Joe Bondy-Denomy
Ivo Gomperts Boneca
John Dallas Boyce
Patricia A. Bradford
Axel A. Brakhage
Stephane Bretagne
William J. Britt
Michael A. Brockhurst
Pierre A. Buffet
Swaine L. Chen
John M. Coffin
Stephen Coleman
Vaughn S. Cooper
Thelka Cordes
Fevzi Daldal
James B. Dale
Blossom Damania
Denise Dearing
Tanneke den Blaauwen
Eric Y. Denkers
Lars E. P. Dietrich
Stephen P. Diggle
Daniel DiMaio
Maxwell Dow
Nisha Duggal
Lothar Elling
Jorge C. Escalante-Semerena
Ariberto Fassati
Edward J. Feil
Alain Filloux
Woodward W. Fischer
Suzanne M. J. Fleiszig
Claire M. Fraser
Clay Fuqua
Jorge E. Galan
Robert F. Garry
Maria Laura Gennaro
Mimi Ghosh
Deanna L. Gibson
Steven R. Gill
Benjamin Glick
Vernita Gordon
Sabina Gorska
Heinrich Georg Gottlinger
Jean Gruenberg
Angelika Gründling
Henk P. Haagsman
Maria Hadjifrangiskou
Susan Hafenstein
Neal D. Hammer
David Harrich
Rasika Harshey
Thomas Hawn
Gerald L. Hazelbauer
Sophie Helaine
Andrew J. Henderson
Hubert Hilbi
Deborah A. Hogan
Kerwyn Casey Huang
Kelly T. Hughes
David A. Hunstad
Urs Jenal
David Kadosh
Ganjam V. Kalpana
Fatah Kashanchi
Daniel B. Kearns
Brian Kelsall
Bruce S. Klein
Manuel Kleiner
Theresa M. Koehler
Julia Ruth Köhler
Eugene V. Koonin
Peter Kraiczy
Oscar P. Kuipers
Kim Lewis
Shan-Lu Liu
Joseph Lutkenhaus
Frank Maldarelli
Harmit S. Malik
David M. Margolis
Adam Martiny
Leonard Mindich
Harry L. T. Mobley
Karl Münger
Kenneth H. Nealson
James D. Oliver
Beth Orcutt
Eric Oswald
Melanie Ott
Bernhard O. Palsson
Laila Partida-Martinez
Thomas Pietschmann
Alexander Ploss
Paul B. Rainey
Timothy D. Read
David A. Relman
Jyothi Rengarajan
Kyu Young Rhee
Andrew Rice
Jason W. Rosch
Gian Maria Rossolini
Monica J. Roth
Manish Sagar
Jason W. Sahl
Linda J. Saif
Álvaro San Millan
Saumendra N. Sarkar
Karla J. F. Satchell
Jeffrey W. Schertzer
Barbara Shacklett
William M. Shafer
Lilach Sheiner
David R. Sherman
Chiaho Shih
Robert F. Siliciano
Viviana Simon
Eric P. Skaar
David Skurnik
Mark S. Smeltzer
David G. Thanassi
Alfredo G. Torres
Ana Traven
M. Stephen Trent
Rodney K. Tweten
Susana T. Valente
Henny C. van der Mei
Willem Van Schaik
David Wang
Scott C. Weaver
Robert T. Wheeler
Alex Wilson
Hui Wu
Jae-Hyuk Yu
Egija Zaura
Zhi-Ming Zheng
Jingen Zhu

